# Characteristics of circulating adaptive immune cells in patients with colorectal cancer

**DOI:** 10.1038/s41598-022-23190-0

**Published:** 2022-10-28

**Authors:** Longyi Zhang, Xuya Chen, Shujin Zu, Yan Lu

**Affiliations:** 1 Clinical Laboratory, DongYang People’s Hospital, 60 West Wuning Road, Dongyang, 322100 Zhejiang China; 2 Reproductive Medicine Center, DongYang People’s Hospital, 60 West Wuning Road, Dongyang, 322100 Zhejiang China

**Keywords:** Gastrointestinal cancer, Tumour immunology

## Abstract

Adaptive immune cells prevent solid tumor progression by targeting and killing tumor cells. However, there are no comprehensive studies on peripheral circulating adaptive immune cell characterization in colorectal cancer (CRC) patients or the effect of tumor-node-metastasis (TNM) stages on these cells. In this study, the number, phenotype, and function of different subsets of circulating adaptive immune cells in peripheral blood of CRC patients were analyzed. We found remarkable differences in CRC patients compared with those in healthy controls, including reduced absolute counts of total T cells, helper T lymphocytes (Th), cytotoxic T lymphocytes (Tc), and double-negative T lymphocytes, a decreased proportion of INF-γ^+^ cells in total T cells and Th, and increased percentages of B cells, plasmablasts, and activated T cells. Compared with early-stage CRC patients, advanced-stage CRC patients showed more severe immunosenescence, which manifested as decreased proportions of CD8^+^ naive T cells with strong proliferative ability and CD8^+^ central memory T cells with immune surveillance function. Proportions and absolute counts of CD8^+^ and CD4^+^ terminally differentiated effector memory T cells were increased, indicating immunosenescence. The immune cell characteristics analyzed in this study serve as a starting point for further research to determine potential clinical implications.

## Introduction

Immune surveillance failure is known to lead to the development of cancer. Adaptive immune cells target and kill tumor cells by interacting with solid tumors during tumor development and progression^1^. The role of different immune cell subsets in promoting or inhibiting antitumor immune responses has been previously described^2–4^. For example, the massive infiltration of CD8^+^ T cells in renal cell carcinoma is considered to be a poor prognostic factor^5^. Among the helper T (Th) cell subsets, patients with high Th17 expression had a poorer prognosis, while those with high Th1 expression had prolonged disease-free survival^6^. Furthermore, increased tumor-infiltrating B cells are associated with a worse prognosis in breast cancer patients^7^. These immunology-based studies were mostly focused on the tumor microenvironment (TME). However, it is evident that studying the TME alone is insufficient to fully understand tumor immunity, since the peripheral circulating immune system is an indispensable component of immune regulation in the body^8^. Additional peripheral immunophenotyping is also required, as this provides the chance to identify adaptive immune cell subsets that are altered in patients with solid tumors^9^, laying the groundwork for a more in-depth investigation of the antitumor immune response mechanism.

Recent studies of colorectal cancer (CRC) have shown that adaptive immune responses in the TME are involved in regulating the balance between CRC cell invasion and body defense^10–13^. However, only a few studies have demonstrated the characterization of peripheral circulating adaptive immune cells in CRC patients, mainly focusing on the characteristics of T cell subsets^14–16^. Furthermore, comprehensive studies on the effect of tumor-node-metastasis (TNM) stages on circulating adaptive immune cells are scarce. In this study, we analyzed the number, phenotype, and function of different subsets of circulating adaptive immune cells in peripheral blood to gain insight into the changes that occur to these immune cells during the progression of CRC.

## Results

### Participants’ characteristics

To study the number and phenotype of T and B lymphocytes, we prospectively recruited 107 patients with “colorectal mass”, 78 of which were included in this study after they were screened for CRC (Supplementary Fig. S1). As summarized in Table 1, there were no significant differences in age and sex between the healthy control and CRC groups. In accordance with TNM staging, the CRC group was further divided into early- and advanced-stage groups, including 36 and 42 patients, respectively. In addition, 20 patients who met the inclusion criteria for the CRC group and 28 healthy controls were studied for interferon-gamma (INF-γ) secretion function of T lymphocyte subsets.

**Table 1:** Basic characteristics of healthy controls and CRC patients to study the number and phenotype of T and B lymphocytes.

Characteristics	Healthy controls (N = 63)	CRC group (N = 78)	*P* value
Age (years)	63 (56–69)	64.5 (57–72)	0.169
**Sex**			0.639
Male	34 (54.0%)	39 (50.0%)	
Female	29 (46.0%)	39 (50.0%)	
**Drinking history**			0.078
Yes	14 (22.2%)	28 (35.9%)	
No	49 (77.8%)	50 (64.1%)	
**Smoking history**			0.272
Yes	12 (19.0%)	21 (26.9%)	
No	51 (81.0%)	57 (73.1%)	
**Tumor site**			
Colon		31 (39.7%)	
Rectum		47 (60.3%)	
**TNM stage**			
Stage I–II		36 (46.2%)	
Stage III–IV		42 (53.8%)	

CRC, Colorectal cancer; TNM, tumor-node-metastasis.

### T lymphocyte subsets in the healthy control and CRC groups

Supplementary Table S1 lists the proportions and absolute counts of T lymphocyte subsets in the healthy control and CRC groups.

**Figure 1:**
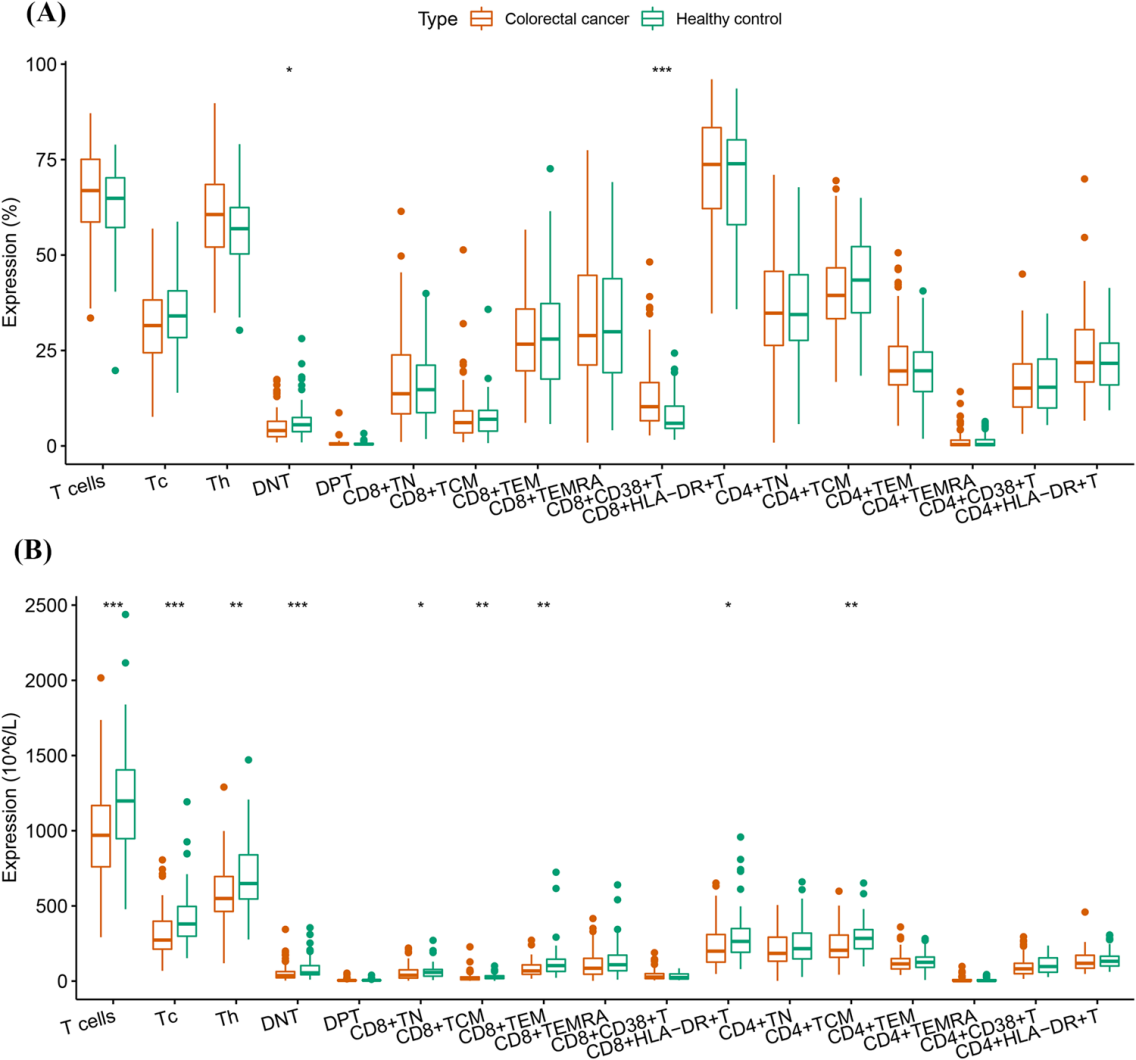
Differences in T lymphocyte subsets between the healthy control and CRC groups. (**A**) Proportions of T lymphocyte subsets. (**B**) Absolute counts of T lymphocyte subsets.

Compared with those in the healthy control group, double-negative T (DNT) lymphocytes (% of T cells) were significantly lower in the CRC group (4.0 [2.4–6.5] vs. 5.5 [3.6–7.6], *P* = 0.018), whereas CD8^+^CD38^+^ activated T cells (% of Tc) were significantly higher (10.3 [6.6–16.6] vs. 5.9 [4.6–10.5], *P* < 0.001) (Fig. 1A). Supplementary Fig. S2a shows the differences in the distribution of T cell subsets in the healthy control and CRC groups.

When the absolute counts of subsets were compared between the healthy control and CRC groups, T cells, namely cytotoxic T cells (Tc) (273.2 [212.2–399.6] vs. 380.4 [296.7–498.9], *P* < 0.001); Th (549.2 [461.8–696.3] vs. 649.0 [537.9–841.8], *P* = 0.008); DNT (36.8 [21.7–63.5] vs. 54.7 [41.8–106.4], *P* < 0.001); CD8^+^ naive T cells (TN) (38.8 [19.8–74.9] vs. 58.1 [32.5–78.4], *P* = 0.038); CD8^+^ central memory T cells (TCM) (17.4 [9.1–28.3] vs. 25.3 [16.0–35.5], *P* = 0.003); CD8^+^ effector memory T cells (TEM) (68.3 [44.5–108.3] vs. 103.5 [61.7–146.6], *P* = 0.003); CD8^+^HLA-DR^+^ T cells (199.2 [125.6–311.6] vs. 263.8 [188.3–359.3], *P* = 0.011); and CD4^+^ TCM (205.0 [157.9–306.0] vs. 284.4 [215.1–343.0], *P* = 0.003) were significantly decreased in the CRC group (Fig. 1B).

### B lymphocyte subsets in the healthy control and CRC groups

We analyzed the proportions and absolute counts of B lymphocyte subsets in the healthy control and CRC groups (Supplementary Table S1).

**Figure 2:**
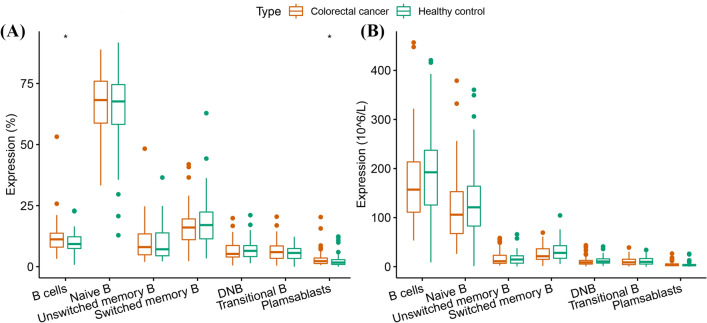
Differences in B lymphocyte subsets between the healthy control and CRC groups. (**A**) Proportions of B lymphocyte subsets. (**B**) Absolute counts of B lymphocyte subsets.

The increase in B lymphocytes (% of lymphocytes) and plasmablasts (% of B cells) in the CRC group was statistically significant (11.2 [8.0–13.7] vs. 9.3 (7.4–12.3), *P* = 0.042 for B lymphocytes; 2.3 (1.3–3.6) vs. 1.7 (0.9–3.1), *P* = 0.016 for plasmablasts) (Fig. 2A). Supplementary Fig. S2b exemplifies the differences in the distribution of B cell subsets in the healthy control and CRC groups. Compared with those of the healthy controls, none of the absolute values of B lymphocyte subsets in the CRC group showed statistically significant changes (Fig. 2B).

### T lymphocyte subsets in the early- and advanced-stage groups

**Figure 3:**
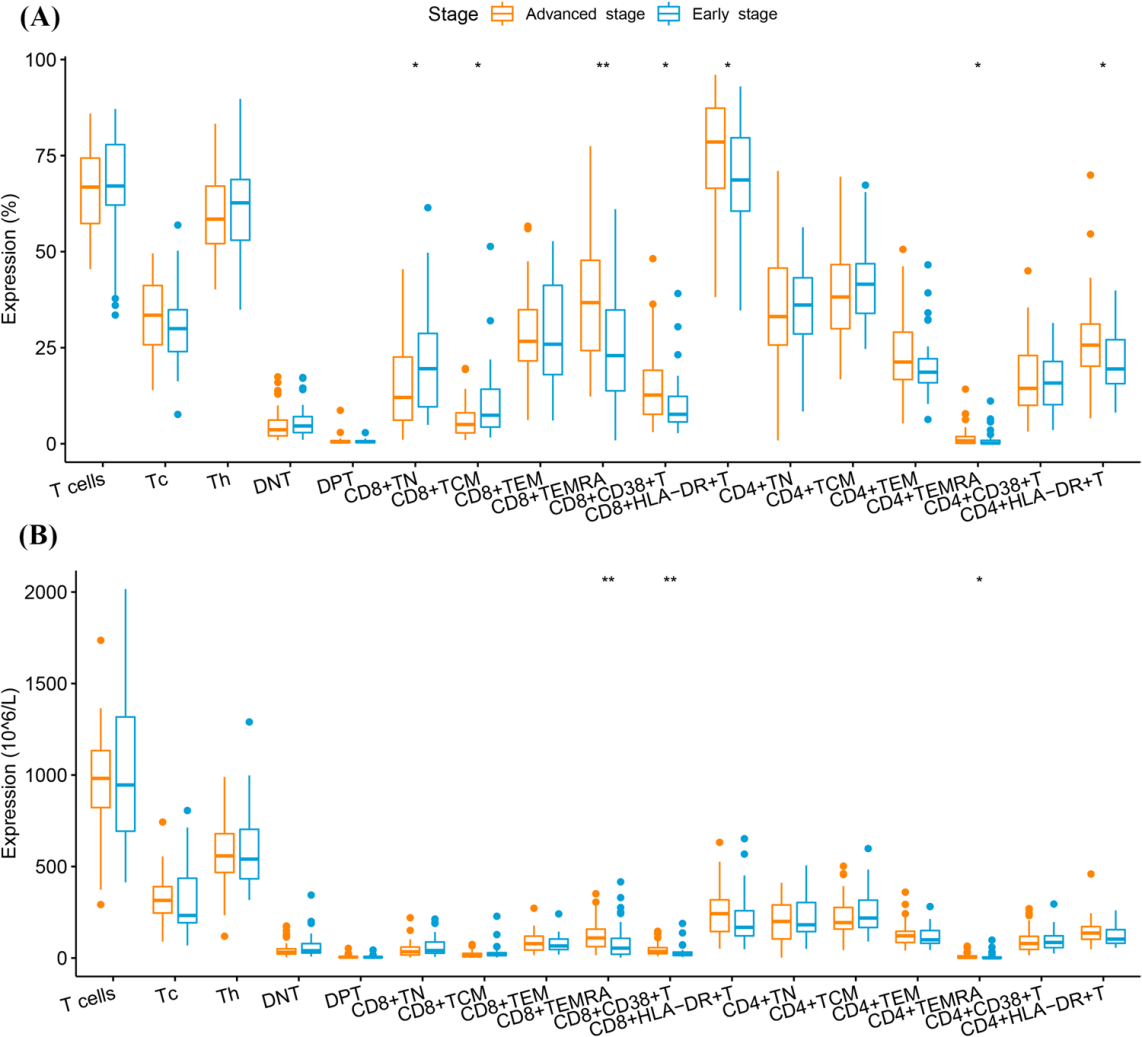
Differences in T lymphocyte subsets between the early- and advanced-stage groups. (**A**) Proportions of T lymphocyte subsets. (**B**) Absolute counts of T lymphocyte subsets.

Regarding the proportions of T cell subsets, a significant decrease in CD8^+^ TN (% of Tc) (12.1 [6.1–22.8] vs. 19.5 [9.5–28.8], *P* = 0.032) and CD8^+^ TCM (% of Tc) (5.0 [2.8–8.1] vs. 7.4 [4.3–14.5], *P* = 0.013) was observed in the advanced-stage group, while CD8^+^ terminally differentiated effector memory T cells (TEMRA) (% of Tc) (36.7 [24.1–47.9] vs. 22.9 [13.2–34.8], *P* = 0.002); CD8^+^CD38^+^ T (% of Tc) (12.7 [7.7–19.2] vs. 7.7 [5.6–12.4], *P* = 0.010); CD8^+^HLA-DR^+^ T (% of Tc) (78.5 [66.1–87.6] vs. 68.7 [60.2–79.7], *P* = 0.033); CD4^+^ TEMRA (% of Th) (0.8 [0.1–1.9] vs. 0.2 [0.1–0.9], *P* = 0.042); and CD4^+^HLA-DR^+^ T (% of Th) (25.7 [20.1–31.2] vs. 19.5 [15.4–27.2], *P* = 0.020) were significantly increased (Fig. 3A and Supplementary Table S2). Supplementary Fig. S2c presents the differences in the distribution of T cell subsets in the early- and advanced-stage groups.

In addition, comparing the absolute counts of the subsets revealed significant differences in CD8^+^ TEMRA, CD8^+^CD38^+^ T, and CD4^+^ TEMRA between the early- and advanced-stage groups (Fig. 3B and Supplementary Table S2) (109.4 [60.6–158.4] vs. 54.1 [20.5–109.0], *P* = 0.006; 35.5 [22.8–57.2] vs. 19.7 [13.7–33.5], *P* = 0.003; and 3.4 [0.8–12.2] vs. 1.2 [0.3–5.2], *P* = 0.048, respectively).

### B lymphocyte subsets in the early- and advanced-stage groups

**Figure 4:**
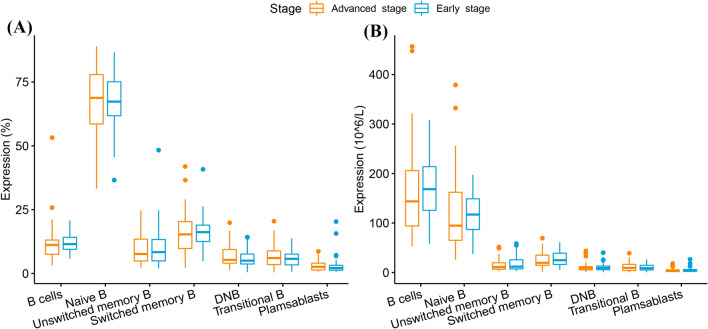
Differences in B lymphocyte subsets between the early- and advanced-stage groups. (**A**) Proportions of B lymphocyte subsets. (**B**) Absolute counts of B lymphocyte subsets.

There were no significant differences (*P* > 0.05) between the early- and advanced-stage groups in the proportions and absolute counts of all B lymphocyte subsets (Fig. 4 and Supplementary Table S2).

### INF-γ secretion function of lymphocytes in healthy control group versus CRC group as well as early-stage group versus advanced-stage group

We further investigated the INF-γ secretion function of lymphocytes in each group (Table 2). Compared with the healthy control group, CD3^+^INF-γ^+^ cells (% of T cells) and CD4^+^CD8^−^INF-γ^+^ cells (% of Th) were significantly decreased in the CRC group (25.8 [18.1–43.7] vs. 43.4 [33.3–52.8], *P* = 0.004; 13.6 (8.9–18.4) vs. 21.5 (15.5–28.0), *P* = 0.004). In addition, CD3^+^INF-γ^+^ cells (% of T cells), CD4^+^CD8^−^INF-γ^+^ T cells (% of Th) and CD4^−^CD8^+^INF-γ^+^ T cells (% of Tc) were compared between the early- and advanced-stage groups, and there was no significant difference.

**Table 2:** The INF-γ secretion function of lymphocytes in healthy control group versus CRC group as well as early-stage group versus advanced-stage group.

Variables	Healthy control group versus CRC group			Early-stage group versus advanced-stage group		
	Healthy control group (n = 28)	CRC group (n = 20)	*P*-value	Early-stage group (n = 7)	Advanced-stage group (n = 13)	*P*-value
Age (years)	59.5 (58.5–70)	65 (58.5–70)	0.571	66 (47–71)	65 (59–68)	1.000
Sex (Male/Female)	13 (46.4%)/15 (53.6%)	14 (70%)/6 (30%)	0.105	4 (57.1%)/3 (42.9%)	10 (76.9%)/3 (23.1%)	0.357
CD3^+^INF-γ^+^ cell, % of T cells	43.4 (33.3–52.8)	25.8 (18.1–43.7)	0.004	23.5 (19.4–39.2)	26.5 (15.3–44.9)	0.905
CD4^+^CD8^-^INF-γ^+^ T, % of Th	21.5 (15.5–28.0)	13.6 (8.9–18.4)	0.004	12.7 (9.9–18.2)	14.5 (7.9–18.7)	0.968
CD4^-^CD8^+^ INF-γ^+^ T, % of Tc	73.0 (67.3–84.1)	68.5 (44.9–82.7)	0.149	64.8 (62.7–82.6)	72.3 (43.0–82.9)	0.501

CRC, Colorectal cancer; INF-γ, interferon-gamma.

## Discussion

Cancer patients are known to exhibit impaired adaptive immune responses because of immunosuppression, and our study confirm this. In this study, we observed significant changes in the circulating adaptive lymphocyte profile of CRC patients compared with those of healthy patients. Furthermore, we assessed differences in the distribution of adaptive immune cells in patients with early- and advanced-stage CRC. As expected, advanced-stage CRC patients showed more severe immunosenescence.

Our study demonstrated a marked reduction in the absolute numbers of total T cells and various T cell subsets in the CRC group, which is consistent with prior research^17–19^. The T cell subsets defined as having different proliferative abilities, lymphatic homing, and effector functions have been well established in previous studies^20, 21^. Tc cells are protective immune cells that directly target tumor cells^22^. Th cells help maintain Tc cell immune responses and regulate the development of Tc cells into functional cells^23^. In peripheral circulation and tissues, both are capable of dynamic metabolic control^24^. The decrease in cell number in CRC patients could be due to a reduction in peripheral circulation produced by adaptive immune cells migrating to tumor tissues. Tc and Th cell depletion and dysfunction, which manifest as a decrease in proliferative and functionally active T cells and an increase in senescent T cells, result from persistent metabolic disturbances directed by tumor cells^25^. This is consistent with our findings in patients with advanced CRC. On one hand, the proportions of CD8^+^ TNs with high proliferative capacity and CD8^+^ TCMs with immune surveillance function dramatically decreased as the tumor progressed. On the other hand, CD8^+^ TEMRA and CD4^+^ TEMRA, both of which are markers of immunosenescence, presented higher proportions and absolute counts, showed lower proliferative potential, and were anti-apoptotic. Hence, antitumor immunity was directly suppressed in advanced-stage CRC patients.

There is no consensus regarding the distribution of B cells in CRC patients. Waidhauser et al.^26^ discovered that B cell counts were lower in CRC patients, whereas Spacek et al.^27^ discovered that B cell counts were higher in colon cancer patients than those in patients in control groups. B cells contribute to cancer regulation primarily through indirect inhibition of T cell reactivity, resulting in antitumor immunosuppression^28^. In this study, B cells were subjected to exhaustive immunophenotyping, revealing that the proportion of peripheral circulating B lymphocytes in CRC patients increased with no significant difference in absolute counts. In addition, there were no significant differences in the percentage and absolute value changes of B cell subsets in advanced-stage CRC patients compared with those of patients in the early stage. However, an increasing number of studies has shown that cytokines secreted by B cells have dual roles in tumors during tumorigenesis and progression^29–31^. Whether the role of B cell subsets in CRC can be further explored from the perspective of cytokine secretion remains to be confirmed by further studies.

CD38 and HLA-DR expression is linked to cellular activation during anticancer immune responses, particularly in Tc cells^32^. Colorectal cancer patients had a higher proportion of CD8^+^CD38^+^ T cells than healthy controls. Simultaneously, the proportion and absolute value of activated Tc cells (CD8^+^HLA^-^DR^+^ and CD8^+^38^+^ T) in advanced-stage CRC patients were higher than those in patients at the early stage. Consequently, T cells may be continuously activated along with malignancy incidence and progression.

In previous studies, the immune status of CRC patients has rarely been comprehensively analyzed in combination with the number, phenotype, and function of adaptive immune cells. In addition to analyzing the number and phenotype of T and B cells, this study also revealed significant impairment of T cell and Th cell subset function in CRC patients by lymphocyte INF-γ secretion function tests, which positively correlates with T cell activation, chemotaxis, and cytotoxicity^33^. Although there is no significant difference in INF-γ secretion between early- and advanced- stages of CRC, impaired immune status is not limited to suppression of immune cell function. A comprehensive analysis of the number, phenotype, and function of immune cells is necessary because patient with reduced immune cell numbers may be more severely immunosuppressed between two patients with no difference in immune cell function^34^.

We performed in-depth immunophenotyping of peripheral circulating adaptive immune cells using flow cytometry. We demonstrated that, compared with healthy controls, CRC patients exhibit remarkable alterations in circulating adaptive lymphocyte subsets. In addition, advanced-stage CRC patients showed a more severe immunosenescence. However, this was a single-center study, and the number of patients was limited. Thus, the results should be further confirmed in a large-sample multicenter study. The immune cell characteristics analyzed in this study therefore serve as a starting point for further research to determine potential clinical implications.

## Methods

### Study population

In this study, we prospectively enrolled patients with a “colorectal mass” found via colonoscopy at Dongyang People’s Hospital. The patients who were included in this study met the following inclusion criteria: (1) colorectal adenocarcinoma confirmed through histopathological results, (2) age > 18 years, (3) absence of surgery and chemoradiotherapy, and (4) complete clinical data. The exclusion criteria were as follows: (1) human immunodeficiency, chronic hepatitis C virus infections, or autoimmune diseases, (2) other primary tumors, and (3) serious infectious diseases. According to the 8^th^ edition of the TNM stage classification criteria developed by the American Joint Committee on Cancer (AJCC), patients were divided into early (TNM stages I–II) and advanced (TNM stages III–IV) stages. In addition, healthy adults were recruited as healthy controls. During the data analysis process, the personal information of the patients was not identifiable. This study was approved by the Ethics Committee of Dongyang People’s Hospital, and all participating patients provided written informed consent. The study was performed according to the guidelines of the Declaration of Helsinki.

### Data collection

Age, gender, smoking history, and alcohol-use history were among the basic data collected from CRC patients and healthy controls. The CRC patients also provided information regarding their TNM stage and tumor site. Patients were considered to have a smoking or drinking history if they had ever smoked or consumed alcohol.

### Lymphocyte phenotype analysis

Fresh peripheral whole blood samples were collected from patients before surgery. In a 10-color flow cytometer (Navios; Beckman Coulter, USA), two staining panels were designed for monoclonal fluorescent antibody labeling of circulating T and B cells (Supplementary Table S3). The procedure for surface staining was as recommended by the reagent manufacturer (Beckman Coulter, USA, and BioLegend, USA). In brief, 100 µL of whole blood was incubated with premixed antibodies for 15 min at room temperature in the dark. Subsequently, OptiLyse C lysis buffer (Beckman Coulter) was added to fully lyse the red blood cells. The flow cytometer matching buffer was then added to the test tube, centrifuged, and washed, and finally resuspended for flow cytometry analysis. Data were analyzed using Kaluza software (version 2.0, Beckman Coulter). The gating strategy is illustrated in Supplementary Fig. S3.

For the T cell panel: Total T lymphocytes were identified through the presence of CD3. After which, according to the expression of CD4/CD8, total T cells were divided into CD4^+^CD8^−^ Th, CD4^−^CD8^+^ Tc, CD4^−^CD8^−^ DNT, and CD4^+^CD8^+^ double-positive T (DPT) lymphocytes. Th and Tc cells were further characterized according to their effector memory differentiation status: TN (CD45RA^+^CCR7^+^CD28^+^CD27^+^), TCM (CD45RA^−^CCR7^+^CD28^+^CD27^+/−^), TEM (CD45RA^−^CCR7^−^CD28^+/−^CD27^+/−^), and TEMRA (CD45RA^+^CCR7^−^CD28^−^CD27^−^). Activated T cells included CD38^+^ or HLA-DR^+^ T cells.

For the B cell panel: B lymphocytes are defined by CD19 or CD20 positivity. According to the expression of CD27/IgD, they are divided into IgD^+^CD27^−^ naive B cells, IgD^−^CD27^−^ double-negative B cells, and unswitched (IgD^+^CD27^+^) and switched (IgD^−^CD27^+^) memory B cells. Additionally, transitional B cells (CD24^high^CD38^high^) and plasmablasts (CD24^−^CD38^high^) were identified based on CD24/CD38.

### Lymphocyte function analysis

Heparinized peripheral blood was used for PMA/ionomycin-stimulated lymphocyte function assays. The study methods were conducted according to the study procedure of Ying Luo et al.^35^ The process can be briefly described as follows: (1) 100 µl of whole blood was diluted with 400 µl of RPMI-1640 medium in 12 × 75 mm polystyrene round-bottom tubes with caps (Falcon 352054, Becton Dickinson); (2) The diluted whole blood was stimulated with 1 µl Cell Stimulation Cocktail (eBioscience, 00-4970-93, including Phorbol 12-Myrisate 13-Acetate 40.5 µmol/L, lonomycin 670 µmol/L) and 1 µl Protein Transport Inhibitor Cocktail Leukocyte (eBioscience, 00-4980-93, including Brefeldin A 5.3 mmol/L and Monensin 1 mmol/L) for 4 h at 37 °C with 5% CO_2_; (3) Premixed monoclonal antibodies (anti-CD45, anti-CD3, anti-CD4, anti-CD56, and anti-CD8) were added for cell surface staining; (4) After the cells were fixed and permeated, anti-INF-γ antibody was added for intracellular staining; (5) Analysis was performed with flow cytometry. Specific information on monoclonal antibodies is shown in Supplementary Table S3. The function of total T lymphocytes was defined as the percentage of CD3^+^INF-γ^+^ cells in T cells. The function of Th was defined as the percentage of CD4^+^CD8^-^INF-γ^+^ T cells in CD4^+^ T cells. The function of Tc was defined as the percentage of CD4^-^CD8^+^INF-γ^+^ T cells in CD8^+^ T cells.

### Statistical analysis

We determined the proportion of adaptive lymphocyte subsets using flow cytometry. A hematology analyzer (XN-9000; Sysmex, Japan) was used to determine total lymphocyte number. Absolute counts for each subset were calculated using the proportions and absolute numbers of lymphocytes.

Statistical data analysis was performed using STATA software (version 14.0), and graphs were visualized using R software (version 4.1.0). The Chi-squared and Wilcoxon Rank Sum tests were used to compare group differences in univariate categorical and continuous variables, respectively. Statistical significance was set at *P* < 0.05.

## Supplementary Information

Supplementary Information.

## Data Availability

All data generated or analyzed during this study are included in this published article.
