# Supplementary material for: Characteristics of circulating adaptive immune cells in patients with colorectal cancer
Source: Sci Rep. 2022 Oct 28;12:18166. doi: 10.1038/s41598-022-23190-0 (PMC9616942; doi:10.1038/s41598-022-23190-0)
Supplement: Supplementary file 1 — Supplementary Information. [file 41598_2022_23190_MOESM1_ESM.pdf]

Characteristics of circulating adaptive immune cells in patients with colorectal cancer

Longyi Zhang[1], Xuya Chen[1], Shujin Zu[2], Yan Lu[1*]

[1] Clinical Laboratory, DongYang People's Hospital, 60 West Wuning Road, Dongyang 322100, Zhejiang, China

[2] Reproductive Medicine Center, DongYang People's Hospital, 60 West Wuning Road, Dongyang 322100, Zhejiang, China

***Corresponding author**

E-mail: luyan11219@hotmail.com (YL)

Supplementary Table S1. Expression levels of peripheral circulating T and B cells in the healthy control and CRC groups.

Subset	Healthy control (N = 63)	CRC group (N = 78)	*P*-value
T cells, 10^6/L	1197.9 (939.7-1409.3)	969.6 (757.2-1168.7)	<0.001
Tc, 10^6/L	380.4 (296.7-498.9)	273.2 (212.2-399.6)	<0.001
$CD8^+$ TN, 10^6/L	58.1 (32.5-78.4)	38.8 (19.8-74.9)	0.038
$CD8^+$ TCM, 10^6/L	25.3 (16.0-35.5)	17.4 (9.1-28.3)	0.003
$CD8^+$ TEM, 10^6/L	103.5 (61.7-146.6)	68.3 (44.5-108.3)	0.003
$CD8^+$ TEMRA, 10^6/L	108.7 (68.9-177.0)	85.7 (45.4-151.6)	0.064
$CD8^+$ $CD38^+$ T, 10^6/L	24.0 (14.3-48.3)	27.8 (15.9-50.4)	0.438
$CD8^+$ HLA-DR^+ T, 10^6/L	263.8 (188.3-359.3)	199.2 (125.6-311.6)	0.011
Th, 10^6/L	649.0 (537.9-841.8)	549.2 (461.8-696.3)	0.008
$CD4^+$ TN, 10^6/L	216.4 (143.0-319.5)	184.4 (132.2-292.4)	0.154
$CD4^+$ TCM, 10^6/L	284.4 (215.1-343.0)	205.0 (157.9-306.0)	0.003
$CD4^+$ TEM, 10^6/L	124.9 (91.4-161.4)	114.4 (80.9-149.7)	0.242
$CD4^+$ TEMRA, 10^6/L	2.8 (0.5-9.3)	2.1 (0.6-10.1)	0.709
$CD4^+$ $CD38^+$ T, 10^6/L	97.0 (58.1-154.8)	81.7 (49.2-119.3)	0.130
$CD4^+$ HLA-DR^+ T, 10^6/L	132.4 (100.0-169.1)	118.4 (85.4-172.4)	0.272
DNT, 10^6/L	54.7 (41.8-106.4)	36.8 (21.7-63.5)	<0.001
DPT, 10^6/L	5.6 (4.0-7.3)	4.6 (2.8-7.0)	0.096

B cells, 10^6/L	192.3 (125.6-238.8)	157.0 (110.8-213.9)	0.101
Naive B, 10^6/L	121.0 (82.5-164.4)	106.0 (67.5-154.0)	0.205
Unswitched memory B , 10^6/L	14.6 (7.0-22.4)	11.1 (6.9-23.6)	0.631
Switched memory B , 10^6/L	28.2 (17.0-43.3)	21.3 (15.0-36.5)	0.078
DNB , 10^6/L	10.7 (6.9-16.8)	8.8 (5.3-13.0)	0.087
Transitional B, 10^6/L	9.8 (5.3-16.6)	8.8 (4.3-15.3)	0.494
Plasmablasts, 10^6/L	3.3 (1.8-4.5)	3.3 (2.1-5.8)	0.249
T cells, % of lymphocytes	64.9 (56.8-70.5)	66.9 (58.4-75.2)	0.194
Tc, % of T cells	34.1 (28.3-40.8)	31.5 (24.1-38.4)	0.178
$CD8^+$ TN, % of Tc	14.7 (8.7-21.2)	13.7 (8.4-23.9)	0.781
$CD8^+$ TCM, % of Tc	7.0 (3.9-9.3)	6.1 (3.4-9.2)	0.542
$CD8^+$ TEM, % of Tc	28.0 (17.1-37.4)	26.7 (19.5-36.0)	0.663
$CD8^+$ TEMRA, % of Tc	29.9 (19.1-43.9)	28.9 (21.2-45.1)	0.972
$CD8^+$ $CD38^+$ T, % of Tc	5.9 (4.6-10.5)	10.3 (6.6-16.6)	<0.001
$CD8^+$ HLA-DR^+ T, % of Tc	73.9 (57.9-80.4)	73.7 (62.1-83.4)	0.391
Th, % of T cells	56.9 (50.1-62.8)	60.6 (52.0-68.5)	0.069
$CD4^+$ TN, % of Th	34.4 (27.2-45.6)	34.8 (26.3-46.2)	0.883
$CD4^+$ TCM, % of Th	43.4 (34.4-52.6)	39.4 (33.2-46.7)	0.089
$CD4^+$ TEM, % of Th	19.7 (13.9-24.7)	19.7 (16.0-26.2)	0.373

$CD4^+$ TEMRA, % of Th	0.4 (0.1-1.6)	0.4 (0.1-1.5)	0.936
$CD4^+$ $CD38^+$ T, % of Th	15.4 (9.9-23.0)	15.2 (10.2-21.5)	0.890
$CD4^+$ HLA-DR^+ T, % of Th	21.6 (15.4-27.3)	21.8 (16.7-30.6)	0.232
DNT, % of T cells	5.5 (3.6-7.6)	4.0 (2.4-6.5)	0.018
DPT, % of T cells	0.5 (0.4-0.7)	0.5 (0.3-0.8)	0.724
B cells, % of lymphocytes	9.3 (7.4-12.3)	11.2 (8.0-13.7)	0.042
Naive B, % of B cells	67.7 (58.1-74.6)	68.2 (58.7-76.0)	0.639
Unswitched memory B , % of B cells	7.1 (4.3-13.9)	8.0 (4.8-13.5)	0.538
Switched memory B , % of B cells	17.1 (11.3-23.1)	16.0 (11.0-19.6)	0.292
DNB , % of B cells	6.5 (4.1-8.9)	5.2 (4.0-8.8)	0.380
Transitional B, % of B cells	5.6 (3.3-7.5)	6.0 (3.4-8.6)	0.559
Plasmablasts, % of B cells	1.7 (0.9-3.1)	2.3 (1.3-3.6)	0.016

Supplementary Table S2. Expression levels of peripheral circulating T and B cells in patients with early- and advanced-stage CRC.

Subset	Early-stage CRC (N = 36)	Advanced-stage CRC (N = 42)	*P*-value
T cells, 10^6/L	945.5 (690.8-1333.1)	981.5 (819.4-1136.8)	0.920
Tc, 10^6/L	232.6 (190.6-442.8)	314.9 (244.8-392.1)	0.270
$CD8^+$ TN, 10^6/L	40.8 (25.8-89.2)	34.1 (16.2-61.2)	0.128
$CD8^+$ TCM, 10^6/L	18.4 (12.7-29.9)	15.1 (6.1-26.1)	0.123
$CD8^+$ TEM, 10^6/L	66.3 (45.6-104.5)	78.6 (41.0-121.8)	0.609
$CD8^+$ TEMRA, 10^6/L	54.1 (20.5-109.0)	109.4 (60.6-158.4)	0.006
$CD8^+$ $CD38^+$ T, 10^6/L	19.7 (13.7-33.5)	35.5 (22.8-57.2)	0.003
$CD8^+$ HLA-DR^+ T, 10^6/L	167.5 (121.1-266.0)	242.3 (141.4-319.8)	0.141
Th, 10^6/L	540.2 (425.7-703.7)	557.8 (466.7-684.4)	0.849
$CD4^+$ TN, 10^6/L	181.2 (143.6-308.5)	199.6 (100.0-291.8)	0.703
$CD4^+$ TCM, 10^6/L	218.2 (161.2-317.8)	193.7 (157.9-276.6)	0.218
$CD4^+$ TEM, 10^6/L	99.5 (80.0-150.2)	121.5 (84.1-149.3)	0.440
$CD4^+$ TEMRA, 10^6/L	1.2 (0.3-5.2)	3.4 (0.8-12.2)	0.048
$CD4^+$ $CD38^+$ T, 10^6/L	85.1 (56.1-125.5)	79.7 (46.6-119.3)	0.681
$CD4^+$ HLA-DR^+ T, 10^6/L	103.8 (79.6-163.0)	136.6 (102.5-172.4)	0.113
DNT, 10^6/L	40.0 (26.9-82.9)	31.8 (21.3-50.9)	0.229
DPT, 10^6/L	4.2 (2.8-6.5)	4.8 (2.7-7.7)	0.833

B cells, 10^6/L	168.5 (124.1-214.0)	143.8 (92.2-206.4)	0.326
Naive B, 10^6/L	117.1 (86.3-149.2)	94.7 (65.0-164.9)	0.502
Unswitched memory B , 10^6/L	11.9 (7.2-27.3)	11.1 (6.7-20.0)	0.394
Switched memory B , 10^6/L	24.8 (15.9-39.1)	19.5 (14.5-35.8)	0.176
DNB , 10^6/L	9.2 (5.6-13.1)	8.8 (5.1-12.0)	0.920
Transitional B, 10^6/L	8.8 (4.8-14.7)	9.3 (3.9-16.6)	0.674
Plasmablasts, 10^6/L	3.4 (2.3-6.1)	3.2 (1.9-5.5)	0.779
T cells, % of lymphocytes	67.1 (62.1-78.0)	66.8 (57.3-74.8)	0.515
Tc, % of T cells	30.0 (23.9-35.2)	33.5 (25.7-41.5)	0.094
$CD8^+$ TN, % of Tc	19.5 (9.5-28.8)	12.1 (6.1-22.8)	0.032
$CD8^+$ TCM, % of Tc	7.4 (4.3-14.5)	5.0 (2.8-8.1)	0.013
$CD8^+$ TEM, % of Tc	25.9 (18.0-41.9)	26.7 (21.5-35.0)	0.857
$CD8^+$ TEMRA, % of Tc	22.9 (13.2-34.8)	36.7 (24.1-47.9)	0.002
$CD8^+$ $CD38^+$ T, % of Tc	7.7 (5.6-12.4)	12.7 (7.7-19.2)	0.010
$CD8^+$ HLA-DR^+ T, % of Tc	68.7 (60.2-79.7)	78.5 (66.1-87.6)	0.033
Th, % of T cells	62.7 (52.6-69.1)	58.5 (52.0-67.4)	0.437
$CD4^+$ TN, % of Th	36.1 (28.2-44.6)	33.1 (25.7-46.2)	0.502
$CD4^+$ TCM, % of Th	41.5 (33.9-47.4)	38.2 (29.8-46.7)	0.344
$CD4^+$ TEM, % of Th	18.6 (15.8-22.2)	21.3 (16.7-29.2)	0.164

$CD4^+$ TEMRA, % of Th	0.2 (0.1-0.9)	0.8 (0.1-1.9)	0.042
$CD4^+$ $CD38^+$ T, % of Th	15.8 (10.2-21.4)	14.4 (9.9-23.6)	0.703
$CD4^+$ HLA-DR^+ T, % of Th	19.5 (15.4-27.2)	25.7 (20.1-31.2)	0.020
DNT, % of T cells	4.6 (2.9-7.2)	3.7 (2.0-6.2)	0.107
DPT, % of T cells	0.5 (0.3-0.8)	0.5 (0.3-0.8)	0.924
B cells, % of lymphocytes	11.5 (9.5-14.2)	11.2 (7.5-13.1)	0.326
Naive B, % of B cells	67.3 (61.7-75.2)	68.8 (58.5-78.0)	0.802
Unswitched memory B , % of B cells	8.4 (4.8-14.3)	7.6 (4.8-13.5)	0.711
Switched memory B , % of B cells	16.2 (12.1-18.9)	15.3 (9.7-20.6)	0.638
DNB , % of B cells	5.0 (3.7-7.7)	5.3 (4.0-9.5)	0.403
Transitional B, % of B cells	5.7 (3.3-7.6)	6.1 (3.5-8.9)	0.581
Plasmablasts, % of B cells	2.1 (1.3-3.3)	2.6 (1.4-4.0)	0.515

Supplementary Table S3. Staining panel for monoclonal fluorescent antibody labeling of circulating T, B cells and INF-γ secretion function of lymphocytes.

Fluorochrome	T cell subsets panel			B cell subsets panel			INF-γ secretion function of lymphocytes		
	Marker	Clone	Source	Marker	Clone	Source	Marker	Clone	Source
FITC	CD45RA	clone ALB11	Beckman Coulter	CD20	Clone B9E9	Beckman Coulter	CD8	clone B9.11	Beckman Coulter
PE	CD4	clone 13B8.2	Beckman Coulter	IgD	clone IA6-2	Beckman Coulter	CD56	clone N901	Beckman Coulter
ECD	HLA-DR	clone Immu-357	Beckman Coulter	CD3	clone UCHT1	Beckman Coulter			
PE-Cy5.5	CD28	clone CD28.2	Beckman Coulter	CD24	clone ALB9	Beckman Coulter	CD3	clone UCHT1	Beckman Coulter
PE-Cy7	CD27	clone 1A4CD27	Beckman Coulter	CD27	clone 1A4CD27	Beckman Coulter	CD19	clone J4.119	Beckman Coulter
APC	CCR7	clone G043H7	Biolegend	CD19	clone J4.119	Beckman Coulter	IFN-γ	4S.B3	biloegend
AA700	CD8	clone B9.11	Beckman Coulter						
AA750	CD3	clone UCHT1	Beckman Coulter	CD5	clone BL1a	Beckman Coulter	CD4	SK3	Caprico biotechnologies
PB	CD38	clone LS198-4-3	Beckman Coulter	CD38	clone LS198-4-3	Beckman Coulter			
KRO	CD45	clone J.33	Beckman Coulter	CD45	clone J.33	Beckman Coulter	CD45	clone J.33	Beckman Coulter

FITC, fluorescein isothiocyanate; PE, phycoerythrin; ECD, PE-Texas-Red; PE-Cy5.5, PE-Cyanin 5.5; PC7, PE-Cyanin 7; APC, Allophycocyanin; AA700, APC-Alexa Fluor 700; AA750,APC- Alexa Fluor 750; PB, Pacific Blue; KRO, Krome Orange

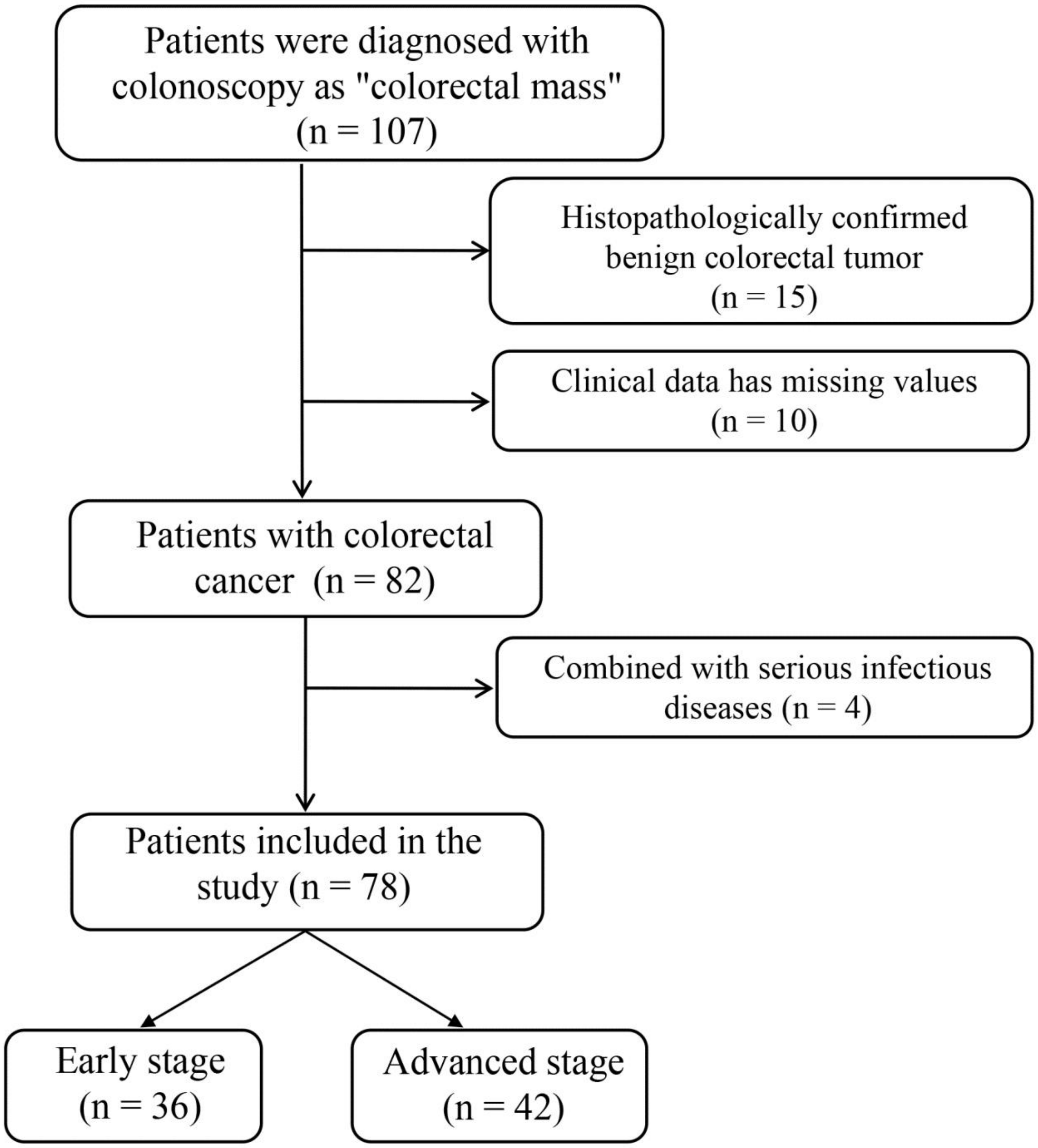

Supplementary Figure S1. Screening process for the patients with CRC to study the number and phenotype of T and B lymphocytes.

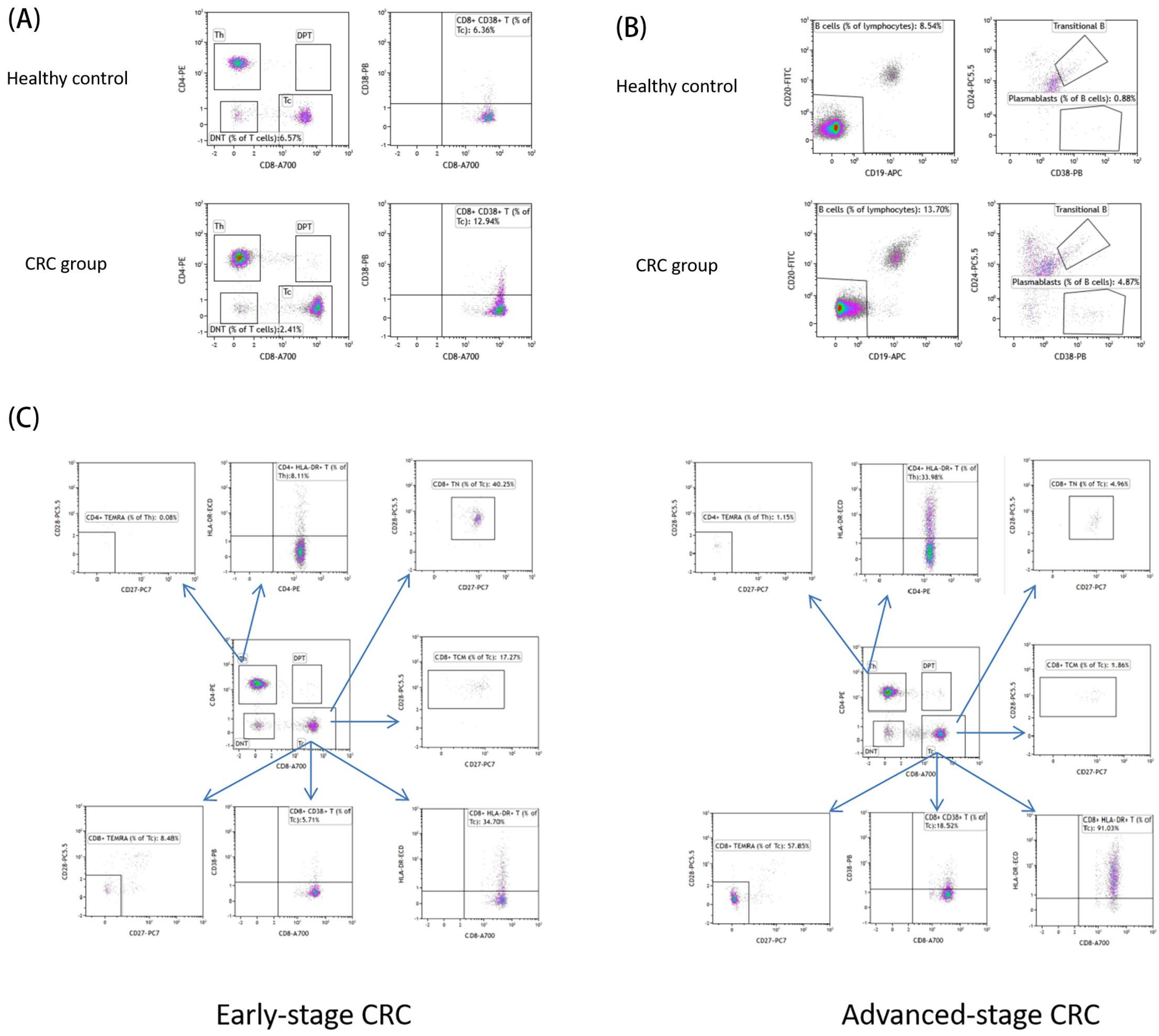

Supplementary Figure S2. The main differences in the healthy control, early-stage and advanced-stage CRC groups. (a) the differences in the distribution of T cell subsets in the healthy control and CRC groups. (b) the differences in the distribution of B cell subsets in the healthy control and CRC groups. (c) the differences in the distribution of T cell subsets in the early-stage and advanced-stage groups.

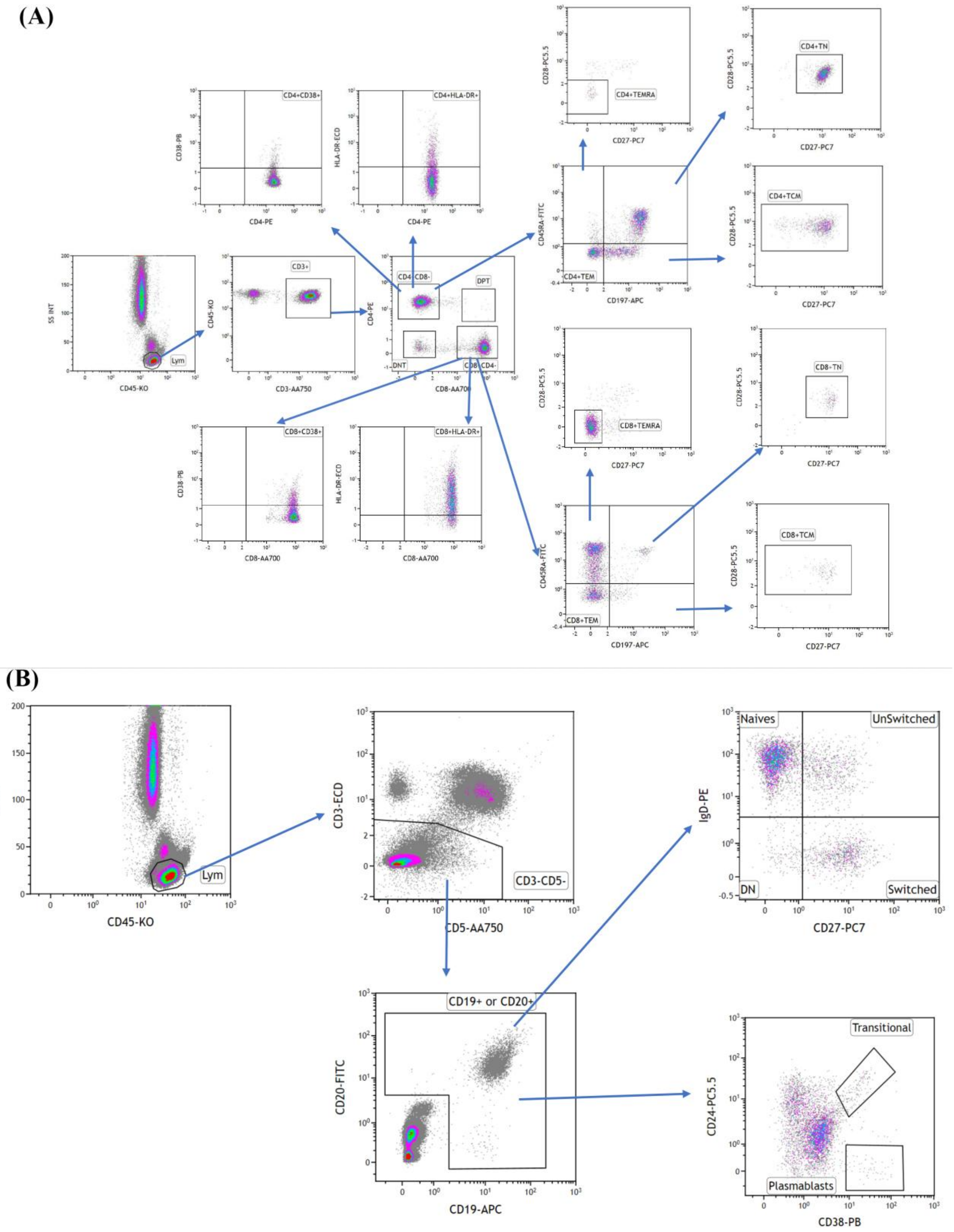

Supplementary Figure S3. Flow cytometry strategy to identify peripheral circulating adaptive immune cells. (a) T cell subsets. (b) B cell subsets.
